# Retracted: Improving Small Intestinal Motility in Experimental Acute Necrotising Pancreatitis by Modulating the CPI-17/MLCP Pathway Using Chaiqin Chengqi Decoction

**DOI:** 10.1155/2021/9873435

**Published:** 2021-08-24

**Authors:** Evidence-Based Complementary and Alternative Medicine

*Evidence-Based Complementary and Alternative Medicine* and the authors have retracted the article titled “Improving Small Intestinal Motility in Experimental Acute Necrotising Pancreatitis by Modulating the CPI-17/MLCP Pathway Using Chaiqin Chengqi Decoction” [1], due to concerns with an undeclared splice identified in Figure 5(a), p-MYPT1, as originally raised on PubPeer [2].

The authors responded to our request for clarification and explained that a band was inappropriately removed from the blot. The authors requested the withdrawal of the article, and with the agreement of the editorial board, the article is being retracted due to the above concerns.
